# Conference Report: COMPASS ’95 TENTH ANNUAL CONFERENCE ON COMPUTER ASSURANCE Gaithersburg, MD June 26–30, 1995

**DOI:** 10.6028/jres.100.045

**Published:** 1995

**Authors:** Bonnie P. Danner, Laura M. Ippolito, Dolores R. Wallace

**Affiliations:** TRW, Government Information Services Division, Fairfax, VA 22033-4417; Computer Systems Laboratory, National Institute of Standards and Technology, Gaithersburg, MD 20899-0001

## 1. Introduction

Cosponsored by the IEEE Aerospace and Electronics Systems Society and the IEEE National Capital Area Council, in cooperation with the British Computer Society, COMPASS (COMPuter ASSurance) is an organization which advances the theory and practice of building computer assurance into critical systems. NIST’s Computer Systems Laboratory hosted the Tenth Annual Conference on Computer Assurance (COMPASS ’95) on June 26–30, 1995, and served as cosponsor with the following industry and government organizations: Arca Systems, Inc.; BDM Engineering Services; Booz-Allen & Hamilton; Computer Associates; CSA; Food & Drug Administration; Intermetrics; Kaman Sciences, Inc.; Logicon, Inc.; Naval Research Laboratory (NRL); Secure Computing Corporation; Space & Naval Warfare Systems Command; SRI International; Systems Safety Society; Trusted Information Systems; and TRW Government Information Services Division. COMPASS ’95 attracted more than 140 participants from government, industry, academia, and foreign countries such as Australia, Canada, Germany, Holland, Israel, Italy, Korea, Sweden, and the United Kingdom. Topics for COMPASS ’95 included testing, analysis and formal development, tools, systems design, standards and processes in the critical areas of safety, reliability, concurrency and real time, fault tolerance, integrity, security, and privacy.

## 2. Tutorials

The conference offered three full-days tutorials, and two half-day tutorials. In the first full-day tutorial, Victoria Stavridou and Pertti Kellomaki, University of London; Andrew Boothroyd and Peter Bradley, AEA; and Timothy Boyce, Jonathon Draper and Robert Smith, GEC Marconi Avionics, presented “The Practical Application of Formal Methods to High Integrity Systems.” This tutorial disseminated the results of the first year and a half of the SafeFM project which provides guidelines on a cost-effective approach to using formal methods in the development and assessment of high integrity systems. The topics presented ranged from specification through development and assessment, unified via the use of a common case study of a fighter aircraft avionics system.

Karen Ferraiolo, Arca Systems, Inc., gave the second full-day tutorial on the “Security Engineering Capability Maturity Model (CMM).” The tutorial provided discussions on security engineering, CMM concepts, the Security Engineering CMM, motivation for its development and use, and the ongoing project to develop a community-accepted model.

In the last full-day tutorial, Michael Evans, Computers & Concepts Associates, discussed “Safety Analysis in the 498 Project Environment.” This tutorial explored the new MIL-STD-498 standard on “Software Development and Documentation,” explored how hazard and product safety analyses can be integrated into the new environments, and looked at the role safety testing, approvals, certification and re-certification play in the process. The attendees developed an understanding of the magnitude of the change that this new standard will cause and how the safety process can be integrated into the project to maximize effectiveness while minimizing cost.

Linda Rosenberg, Unisys, presented the first half-day tutorial on “Metrics for Risk Assessment, Software Quality, and Process Improvement.” This tutorial provided information about a measurement program and showed how to apply the Goal/Question/Metrics paradigm. Project metrics from the NASA Goddard Space Flight Center were used to demonstrate collection, application, interpretation, and benefits.

In the second half-day tutorial, Albert M. K. Cheng, University of Houston, discussed “Real-Time Rule-Based Systems: Analysis & Optimization.” This tutorial presented the basis of the technology for building the next generation real-time environment. Topics covered included structural complexity of rule-based systems, semantics-based static and dynamic response time analysis, parallel rule-based execution, automated optimization and rule-base synthesis, and fault-tolerant rule-based systems.

## 3. General Conference

Bonnie Danner, General Chair, and John Rushby, Program Chair, opened COMPASS ’95 with welcoming remarks. COMPASS ’95 included a tools fair that displayed 13 tools from 11 vendors: *Certify*, Software Engineering Technology, Inc.; *EVES*, Ora Canada; *FEAT* and *INDICT*, CTA, Inc.; *McCabe Testability Tool*, McCabe & Associates; *Penelope*, *Romulus*, and *TableWise*, ORA Corp.; *PiSCES*, Reliable Software Technologies Corp.; *PVS*, SRI International; *RDD-100*, Ascent Logic Corporation; SCR*, NRL; and *Verification Support Environment*, Bundesamt fuer Sicherheit in der Informationstechnik.

Robert N. Veeder, Privacy Advocate of the Internal Revenue Service (IRS), presented the keynote speech on “Information Technology, Its Use and Effect on Privacy.” He described privacy as the right to be left alone, and from a computer privacy perspective, he discussed privacy in terms of information availability, integrity and confidentiality. Mr. Veeder also addressed the National Information Infrastructure (NII) Task Force’s emphasis on privacy as one of its most important concerns. He noted that ensuring security and privacy is a global issue and no one nation can set policy for the rest of the world. At the conclusion of his talk, Mr. Veeder remarked that this is an exciting time to be involved in privacy and security issues. He answered questions from the audience concerning privacy, the NII, and the IRS.

## 4. Testing

Jeffrey Voas, Reliable Software Technologies Corporation (coauthor: Kevin W. Miller, Sangamon State University), presented the first paper of the conference, “Examining Software Quality (Fault-Tolerance) Using Unlikely Inputs: Turning the Test Distribution Upside Down.” He stated that assessing the fault tolerance of rare input states needs to be done for higher software assurance, and discussed an Extended Propagation Analysis algorithm, which concentrates on data state propagation.

Jefferson Offut, George Mason University (coauthor: Zhenyi Jin, George Mason University) discussed “Integration Testing Based on Software Couplings.” He defined twelve coupling levels (levels of testing). The paper presents the results of a Proof of Concept Study and concludes: “coupling based testing works better than category-partition testing.”

Richard Carver, George Mason University (coauthor: Ronnie Durham, Automation Research Systems, Ltd.) presented “Integrating Formal Methods and Testing for Concurrent Programs.” He gave the following conclusions: test constraints provide effective guidance for selecting test sequences; many tradeoffs are involved in writing a formal specification; and test each property in turn, with unrelated events hidden.

## 5. Analysis and Formal Development of Safety-Critical Systems

M. Nicholson, University of York (coauthors: J.A. McDermid, University of York and D.J. Pumfrey, British Aerospace Dependable Computing Systems Centre), described an “Experience with the Application of HAZOP to Computer-Based Systems.” He discussed the application of HAZOP and related techniques to four computer-based systems. The focus was the integration of HAZOP with safety life cycle activities and working practices in the avionics domain.

Glen R. Bruns, University of Edinburgh, presented “Refinement and Dependable Systems.” He applied modal process logic, a generalization of the process algebra CCS, to the verification of an industrial failure-recovery protocol in the air traffic control domain. The main formal, technical result of the study was that the design of a high-level recovery protocol is a valid refinement specification.

S.F.M. van Vlijmen, Utrecht University (coauthor: J.W.C. Koorn, Compuware Technology B.V.) discussed “The Safety Guaranteeing System at Station Hoorn-Kersenboogerd (Extended Abstract).” He described the formal verification of the correctness of computerized control for the safe and timely movement of trains for the Dutch Railway Company. The authors modeled and verified a Vital Processor Interlocking using the process algebraic language muCRL and automated tools.

## 6. Safety Kernels

Paul Ammann, George Mason University, began this session with a paper on “A Safety Kernel for Traffic Light Control.” He observed that one way of looking at security is that security is a special case of safety. So, when looking at safety, choose a successful security method and generalize it. The paper illustrates the use of standard Z specification analysis and includes initialization check, totality checks, and revelation of interesting cases.

Kevin Wicka, University of Virginia (coauthor: John Knight, University of Virginia), presented “On the Enforcement of Software Safety Policies” which summarizes the kernel approach on two case studies (a neurosurgical device and a 2 MW nuclear research reactor). He described a system design that employs user-level safety kernel, closed loop device control, and command authentication with an implementation strategy using a special purpose specification language and safety kernel synthesis.

## 7. Tools for Tabular Formal Specification Methods

This session gave an overview of three formal methods tools. D.N. Hoover, ORA (coauthor: Zewei Chen, ORA), described “Tablewise, a Decision Tool Table.” Using Finite Decision Diagrams, this tool can check for consistency and completeness of tabular specifications. Other capabilities of this tool include Ada/C code generation and generation of English-language specifications from tabular specifications.

Connie Heitmeyer, NRL (coauthors: Alan Bull, Carolyn Gasarch, and Bruce Labaw, NRL), discussed a set of CASE tools, “SCR*: A toolset for Specifying and Analyzing Requirements,” for developing formal requirements specifications expressed in the SCR (Software Cost Reduction) tabular notation. The set includes an editor, a consistency checker, a simulator, and a verifier.

Marsha Chechik, University of Maryland (coauthor: John Gannon, University of Maryland), presented an “Automatic Analysis of Consistency between Implementation and Requirements: A Case Study.” This case study illustrates the use of a tool called Analyzer to check consistency and completeness of an implementation using state transitions specified in the requirements document. The tool verifies safety properties and performs inter-procedural analysis which may involve multiple state machines.

## 8. Application of Formal Methods

This session began with Ricky W. Butler, NASA Langley Research Center (coauthors: James L. Caldwell, Victor A. Carreno, C. Michael Holloway, and Paul Miner, NASA Langley Research Center and Ben L. DiVito, Vigyan, Inc.) presenting the paper “NASA Langley’s Research and Technology Transfer Program in Formal Methods.” He described the growing need for formal methods in aerospace; a rationale for a solution based upon formal methods; NASA Langley’s program strategy; highlights of some accomplishments; and insights gained.

Wolfgang Reif, University of Karlsruhe (coauthors: Gerhard Schellhom and Karl Stenzel, University of Karlsruhe), discussed “Interactive Correctness Proofs for Software Modules Using KIV.” The Karlsruhe Interactive Verifier (KIV), developed for safety-critical systems, provides formal specification of functionality and safety, and stepwise refinement of specifications.

Grace Hammonds, AGCS (coauthors: Randall W. Lichota, Hughes Technical Services Company; Geoffrey Hird, Odyssey Research Associates; and Jack Wool, Arca Systems, Inc.), presented “Command Center Security: Proving Software Correct” which covered the PRISM program and command center security; the application of correctness proofs to increase the level of assurance of security in Air Force systems; the use of Theorem Proving Tools in the Romulus Security Modeling Environment; an example problem; future applications to Fortezza and guard systems; and using belief logic in conjunction with a process model formalism.

## 9. Panel: Safety and Security Issues in Developing and Operating Intelligent Transportation Systems

Dennis Lawrence, LLNL (Moderator), gave an overview of safety and security issues. Frederick D. Cwik, Jr., Senior Engineer Standards and Telecommunications, The Intelligent Transportation Society (ITS) America, described the forces changing surface transportation from the ’60s to the ’90s. He discussed an informal group of industry, university and government representatives, called Mobility 2000, who come together to promote the use of advanced technologies to improve highway safety and efficiency. He presented an overview of the Intermodal Surface Transportation Efficiency Act and then focused on ITS and the Strategic Plan for ITS in the United States.

Bret Michael, California Partners for Advanced Highways and Transit Program, UCLA at Berkeley, discussed the risk issues associated with a cooperative, fully automated highway system (AHS), where all driving tasks are automated. He described three hypothetical scenarios that could lead to safety incidents. He discussed risks and key factors to public acceptance to AHS. He raised many questions and issues associated with implementation of AHS and safety-critical software concerns that must be addressed.

The audience addressed many questions and offered comments on the feasibility of and issues associated with ITS and AHS. Many of these issues remain open; the security and safety challenges for the software intensive systems are formidable.

## 10. Algorithms for Critical Systems

Christof Fetzer, University of California (coauthor: Flaviu Cristian, University of California), presented “An Optimal Internal Clock Synchronization Algorithm.” He proposed an optimal convergence function to achieve fault-tolerant, internal clock synchronization in the presence of arbitrary process and clock failures. A simple, easy-to-compute convergence function bounds the maximum drift rate of a correct hardware clock. He described functional specifics and an overview of the correctness proofs.

Shankar Pal, Pennsylvania State University, discussed “A Locking Protocol for Multi-level Secure Databases Using Two Committed Versions.” He described a locking protocol for multi-level secure databases which produces one-copy serializable and strict schedule, and presented some useful details of snapshot maintenance for locking protocols in secure databases.

## 11. Standards and Processes for Critical Systems

Divya Prasad, University of York (coauthors: John McDermid and Ian Wand, University of York), presented “Dependability, Terminology: Similarities and Differences.” She discussed the conflicting results of her study of terminology used in the critical software community. Terms that provided the most inconsistencies were those used for dependability (availability, safety, security); error, fault, and failure; system and environment; hazard, severity, and risk. The paper discusses the entities and attributes associated with each definition.

Debra Herrmann, Food and Drug Administration, described “A Methodology for Evaluating, Comparing, and Selecting Software Safety and Reliability Standards.” She discussed the results of applying her methodology to several software safety and reliability standards. The criteria for the evaluation included general factors, product characteristics, process characterization, personnel characterization, risk management, and overall standards framework.

Lillian Zelinski, SAIC, presented “Constructing Independent Verification and Validation (IV&V) Life-Cycles Using Process Kernels.” She described IV&V through the use of process kernels throughout the life cycle. The IV&V life cycle methodology is based on a predefined repository of process kernels from which the IV&V life cycle for a given project is defined.

## 12. Formal Verification, Design, and Documentation

Ajin Jirachiefpattana, LaTrobe University (coauthor: Richard Lai, LaTrobe University), reported on “Automated Verification in an Estelle-NPN Based System for Protocol Verification.” He discussed his experience in building and using tools that can automatically verify Estelle specifications which have been translated to Numerical Petri Net (NPN) specifications.

David Parnas, McMaster University (coauthor: Brian James Bauer, McMaster University), presented “Applying Mathematical Documentation: An Experience Report.” He described how a relatively inexperienced and unsophisticated programmer was able to find several errors in the program through this process of documentation. This example supports the position that precise documentation methods can be used by even novice programmers with immediate benefits.

## 13. Evening Events

Amrit Goel, Syracuse University, presented a Birds of a Feather session on “Software Engineering Metrics.” He reviewed the role of metrics as prognosticators of software quality and productivity. He raised a primary question: “How do we determine if the software/system is ready for operational testing?” Some of his overall remarks about metrics addressed proper data analysis and summary presentations as a way to cut down dramatically from original data collected; the use of classification trees and neural networks for process comparisons; and what is going on in research for quality metrics (the state of the art is not very far along). His view is that we don’t have a sound engineering and scientific basis for software predictions yet, but he believes the potential is there for use of statistics in a truly scientific way to support software engineering.

Peter G. Neumann, SRI International, addressed the COMPASS ’95 banquet with a retrospective of prior COMPASS banquet topics and observations of risks to the public in the use of computer systems and related technology. He presented “Risks of the Year” to illustrate where the field stands today. The events receiving this year’s disaster award include the Pentium for its lack of precision, recalls of Windows ’95, lack of National Information Infrastructure security, and the Mitnick security breaches that took officials one year to resolve. Mr. Neumann closed his talk with some thoughts on the future technologic risks and countermeasures. Application of new technologies such as weapons in space and sensors in space will present complex problems without simple solutions. Computers and networks may be subverted easier and faster than the technologies needed to protect them, in part due to the long lead time from research to practice. Learning from our errors and failures, funding for research, and public education are key factors to effectively reducing computer risks today and in the future.

## 14. COMPASS ’96

COMPASS ’96 will be held June 17–21, 1996, at NIST in Gaithersburg, Maryland. The deadline for papers submitted for COMPASS ’96 is January 15, 1996. For information about COMPASS ’96 or how to obtain proceedings of COMPASS ’95, contact Dolores Wallace, Computer Systems Laboratory, National Institute of Standards and Technology, Building 225, Room B266, Gaithersburg, MD 20899-0001; telephone (301) 975-3340 or fax (301) 926-3696.

